# How ventilation behaviour contributes to seasonality in airborne disease transmission

**DOI:** 10.3205/dgkh000414

**Published:** 2022-07-01

**Authors:** Christian Redder

**Affiliations:** 1 Delbag GmbH, Herne, Germany

**Keywords:** air hygiene, natural ventilation, seasonality, airborne diseases, SARS-CoV-2, COVID-19, exposure modelling, dependency effect

## Abstract

User behaviour for natural ventilation is known to be strongly corelated to outdoor temperatures. In areas of moderate climate, this leads to an increased fresh air supply in summer, which reduces the exposure level towards airborne pathogens. Modelling of numerous random exposure situations in household, school and various settings, based on the long-term climate data from Berlin, showed that this effect is likely to contribute significantly to the overall seasonality of airborne diseases.

## Abstract (de)

Es ist bekannt, dass das Verhalten bei der Nutzung manueller Lüftungsmöglichkeiten, wie Fenster und Türen, stark mit der Außentemperatur assoziiert ist. In den gemäßigten Klimazonen führt das zu erhöhter Frischluftzufuhr im Sommer, was die Exposition gegenüber aerosolübertragenen Krankheiten reduziert. Die Modellierung zahlreicher randomisierter Kontaktsituationen in Schul-, Haushalts- und Alltagssettings, basierend auf den Langzeit-Klimadaten der Stadt Berlin, zeigt, dass dieser Effekt signifikant zur Gesamt-Saisonalität aerosolübertragbarer Krankheiten beitragen könnte.

## Introduction

There is a seasonality in the transmission of many airborne diseases. In several macroscopic studies, UV radiation, temperature and other environmental factors, which strongly depend on the meteorological seasons, were found to correlate with the reproduction number [1], [2], [3], [4], [5]. Factors such as strong UV radiation and warm temperatures in summer are obviously not statistically independent from each other, which makes the interpretation of correlations with pathogen transmission difficult. Human behaviour regarding natural ventilation is also strongly related to these environmental factors. In general, the infection probability in outdoor environments is much lower for many diseases that are transmitted though the air, especially for those where increased infectious aerosol concentrations play a role [6], [7], [8]. On the other hand, the direct impact of environmental factors such as weather and UV radiation is strongly reduced inside buildings. This poses the question about the extent to which reduced transmission can be explained by environmental factors per se and how much human behaviour is a driving factor in the seasonality of diseases. An outdoor shift in the contact patterns alone will possibly explain part of the seasonality. However, since even in summer, the transmission of pathogens occurs predominantly in indoor environments, the contribution of the individual’s behaviour within buildings also needs to be investigated. In a recent paper on the factors driving indoor SARS-CoV-2 aerosol exposure, we showed that typical summer ventilation behavior can significantly reduce exposure, compared to typical behavior in cold weather periods [9]. The temperature difference between indoors and outdoors, which mainly drives the air exchange through windows or open doors, is likely to be smaller during summer in most temperate climate zones. However, the opportunity to keep open windows longer or even constantly easily overcompensates this. With outside temperatures close to the range of thermal comfort, there is no reason to prevent air exchange. Warm temperatures and sunny weather may even lead to improved ventilation. Such seasonality in ventilation behaviour for natural ventilation has been shown by many researchers. The outdoor temperature seems to have the strongest impact, showing a linear correlation for a wide range of mean daily temperatures [10], [11]. At very warm temperatures, the effect may stagnate and remain on a high level. A study from China [12], where the total opening time of windows was recorded, suggests that this may happen at daily mean temperatures >26°C. It must be mentioned that the investigated buildings in this study also had mechanical ventilation options, but the mechanical ventilation was not increased significantly above 26°C. Therefore, it can be concluded that there may be a high temperature level at which the linear increase stops. Other detailed studies, which documented the average number of open windows, are from the UK, where the recorded temperatures were lower and thus no stagnation was found [13], [14]. Since the following calculations are based on the climate diagram of Berlin, where high daily mean temperatures are rare, a linear correlation appears to be a reasonable assumption. 

The focus of the present study is to test the hypothesis that seasonal behaviour regarding natural ventilation significantly contributes to the seasonality of airborne diseases. To differentiate this specific effect from others, this hypothesis must be tested using a microscopic approach on the scale of individual contact situations. The accurate quantification of the real effect for a community would require elaborate clustering and parameterization of the model clusters. Since thisinformation is not available, a heuristic approach is indicated in order to show whether the effect is within a relevant range. We thus conducted aerosol-exposure modelling, assuming different levels of additional ventilation due to increasing outdoor temperatures, and calculated the transmitability for three exemplary cluster settings. 

## Methods

The modelling was carried out for a large set of randomized contact situations, with varying room size, ventilation and durations. Each contact situation represents a contact between an infected person and a healthy person. A constant airflow supply is assumed. The model calculates the transmission probability for the healthy person based on the calculated exposure and an exponential dose-response model. To realize this, the calculated exposure is transformed to a quantum dose based on the exposure to a reference situation (15 minutes, 12 m/h³, 60 m³). Infection probabilities of 10% to 90% in the reference situation were assumed, in order to cover a wide range of pathogens.

Since it is difficult to create complete, randomized indoor-contact-patterns for a whole community, three settings with different model parameters were defined. The parameters employed are shown in Table 1 (Tab. 1). The differentiated contact settings are as follows:

1. Household contacts, with medium to very long daily contact periods in small, slightly to moderately ventilated rooms. Most residential buildings have easily accessible natural ventilation possibilities, suggesting that many situations are affected by behaviour.
2. School contacts, with long contact times in medium sized, moderately ventilated rooms. Only 10% of German schools have mechanical ventilation. Therefore, most of the situations are affected.
3. Random contacts people may have at work, leisure activities, shopping etc. During these activities, the number of professionally, mechanically ventilated buildings is probably the highest. Therefore, in more situations, there will be no significant seasonal increase of the ventilation. The number of situations affected by ventilation behaviour is assumed to be lower than in the other settings.

It is likely that for different reasons, some situations are not affected by ventilation behaviour at all. Since no reliable data on this are available, three sets of calculations were conducted. A conservative scenario considered the effect only for 25% of all situations, a less conservative approach for 50% and a third approach considered the effect for 75%. The exact extent of the additional ventilation is also difficult to determine, as many different situations are affected and the literature mostly concerns only very specific settings. Therefore, three sets ranging from a very low mean increase of 10% over medium values of 25% to a high value of 75% were considered. The values considered for each specific situation are defined to have a random variation of ±20% of the mean increase. Based on the long-term climate diagram of Berlin [15], we assumed a maximum additional ventilation at the maximum temperatures in late July, while it is zero at the temperature low point in late January. The trend was implemented by a phase.shifted sine function, which shows a good fit to the data (R²=0.984).

**Figure 1:**
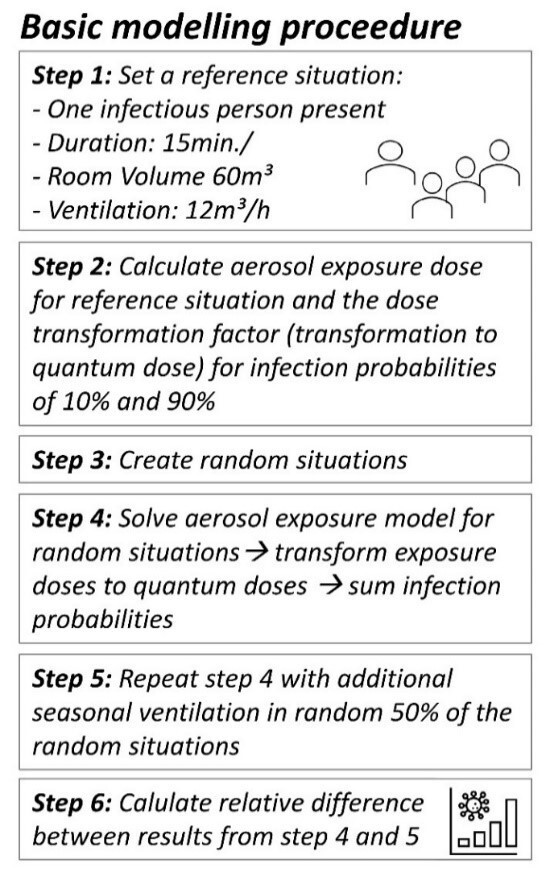
Basic principle of the model used

The aerosol model to calculate the exposure dose was simplified in some points in order to make it more universal and more easily computable. The whole formal description of the model can be found in the following section. To justify the simplifications in the aerosol model, the impact was tested systematically using a more sophisticated model, as suggested in [9]. The first simplification is that the d viability decay of the airborne pathogen is not considered. For a test situation of 60 minutes inside a 60 m³ room, a ventilation rate of 120 m³/h was compared to a situation with 150 m³/h (+25%). The exposure difference with a decay rate of 0 and 2,65*10^–4^*s*^–1^, which is the decay for SARS-COV-2 at moderate humidity according to [16] was compared. The difference in the reduction was 1.4%, with a total reduction of 9.2% with and 10.6% without decay of viability. The second simplification is that the air supply is assumed to be constant, which is probably rarely the case with natural ventilation. Therefore, the same test was repeated, but instead of the viability, the ventilation mode was varied from constant to two short openings. The difference in the reduction was only 0.03%. The results suggest that it is acceptable to neglect these factors. However, for airborne pathogens with high decay rates, the results may overestimate the seasonality effect. Figure 1 (Fig. 1) summarizes the principle of the model.

The calculations compare the isolated effect of reduced exposure due to increased ventilation. All considerations are for airborne transmission, even if not explicitly stated. Taking any kind of immunity could influence the results. Therefore the immunity status is constant for all seasons. The basic reproduction number is

(Equation 1) 

*R*_0_=*τ*
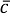
*d*

where *τ* is the average transmission probability per contact, **
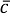
** the average contact rate and *d* the duration of infectivity. When *d* and **
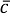
** are deemed constant, *R*_0_ solely depends on *τ*. The infection probability for individual situations can be described by an exponential dose-response model [17]. Since the exact exposure dose cannot be determined, a quantum dose *D_Q_* is used for the quantification:

(Equation 2) 

*f*(*D_Q_*)=1–exp(–*D_Q_*) 

With this dose-response relationship, the average airborne transmission probability for indoor situations can be calculated by the number of indoor contacts, *N_ind_* , that infected persons have and the specific quantum dose of the situation:

(Equation 3) 


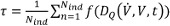


It is assumed that airborne transmission can be modelled by an aerosol model which only calculates the exposure to infective aerosols depending on the room volume *V*, the ventilation air-flow **
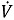
** and the exposure time *t*.

The aerosol model considers the ventilation **
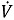
** (assuming ideal mixing), the room volume *V* and the exposure-time *t* and integrates the concentration caused by one infective person over time. Since the real number of pathogens is unknown, a normalized exhalation of 1 pc. per m³ per second is assumed. The assumption of ideal mixing is likely to underestimate the risk of direct short-range transmission. But as the trends from the COVID-19 pandemic suggest, many common diseases have a significant long-range transmission [18] and the results will probably be very valid for the overall effect.

(Equation 4)


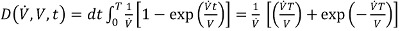


In order to use this exposure dose for the quantum dose model, the calculated dose must be transformed. Equation 4 is thus applied to a reference situation to create a reference exposure:


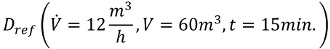


Then Equation 2 is solved for *D_Q_*, so that a specific reference infection probability value for the reference situation can be assigned to *τ*_ref_ and the quantum dose calculated accordingly:

(Equation 5) 

*D_Q–ref_*=–ln(1*–τ_ref_*)

To solve Equation 2 using a specific dose from equation 4 for a specific situation, the results from Equation 4 must be transformed as follows:

(Equation 6)


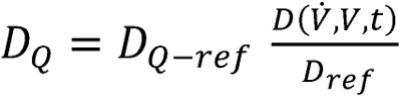


As mentioned before, the long-term climate table of Berlin shows a temperature curve very close to a sine, with a low point in late January and a high in late July. If Δ**
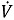
**_ma__x_ [%] is the maximum average increase of the additional air flow and the size of the increase is correlated to a sinusoidal temperature curve with its low point on January 31^st^ und its high point on July 31^st^, the seasonally corrected air flow can be modelled as follows:

(Equation 7)


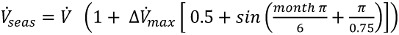


where *month* is the time of the year, *month*=0 is January 1^st^ and *month*=12 is December 31^st^. Accordingly, the average seasonal reduction of the infection probability at a certain time of the year is:

(Equation 8) 

Δ*τ_seas_*=*τ(*
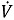
*)–τ(***
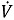
***_seas_ )*

since the additional seasonal air flow described in Equation 7 is understood as a mean value. The extra air flow is distributed with a random variation of ±50% to the randomized situations. This variation, just like the air flow, room size and duration for each setting, are randomized using the Matlab function “rand” with the “Mersenne Twister” algorithm. The algorithm has a period of 2^19937^ and is reset with every new start of the model. Therefore, unwanted correlation can be ruled out [19]. The randomization causes noise in the result, depending on the number of calculated situations and the rate of affected situations. With increasing *N*, the curves converge to the curve of *N*→∞ Since noise is equally distributed around the limit value graph, *N* was chosen in order to achieve optically smooth curves, resulting in ranges between 1,000,000 and 5,000,000.

## Results

The results for the seasonal Δ***t*** values are shown in

- Figure 2 (Fig. 2),
- Figure 3 (Fig. 3),
- Figure 4 (Fig. 4),
- Figure 5 (Fig. 5),
- Figure 6 (Fig. 6),
- Figure 7 (Fig. 7),
- Figure 8 (Fig. 8),
- Figure 9 (Fig. 9), and
- Figure 10 (Fig. 10).

The blue curves show the results for max. +10% additional ventilation, the black curves +25% and the red curves show the results for 75%. In each colour set, the top line represents a disease with a 10% attack rate in the reference situation and the bottom one a 90% attack rate. For all cases, a significant seasonal effect was found. Even for the very conservative assumptions, notable reductions of the transmittability can be shown. The seasonality is stronger for disease with lower attack rates, but the difference is slight.

**Figure 11:**
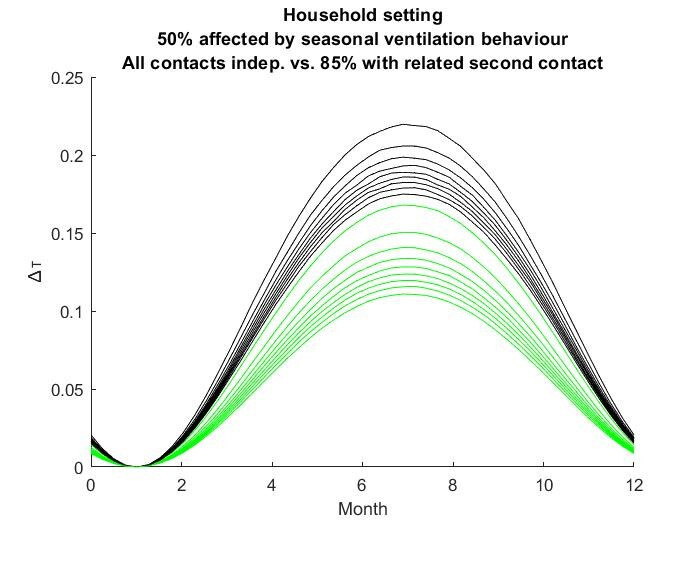
Dependency Effect of consecutive contact situations; the black set of curves is equal to the black set in Figure 3, the green curves shos the same set with reduced ?t due to the dependency effect.

A potential source for an overestimation of the seasonal effect is the independency of contacts within one setting. All modelled random contacts start with an exposure of zero and are therefore independent of each other. When contacts are not independent, the infection risk is dominated by the contact with the highest exposure. If subsequent contacts with the same person have a certain share of the overall contacts, this may affect the results significantly. The household contacts seem the most susceptible setting for this effect. If a couple have dinner in a well-ventilated dining room, the increased ventilation in this situation may not be very relevant if they sleep in the same room with closed windows for hours. In order to quantify the size of the described effect, the original household calculations, which assumed that 50% of all situations were affected by additional natural ventilation with max. average 25% extra ventilation, were extrapolated **as follows:** It was predefined that 85% of all contacts from this set would have a dependent contact situation. For these 85%, the original situations were cut off after 30% to 70% of the time (50% average), and dependent subsequent situations for the remaining duration were created. Except for the duration, the parameters for the subsequent situation were newly created randomly, including the question of whether or not they were affected by seasonal ventilation behaviour. The results are shown in Figure 11 (Fig. 11). A significant reduction of the seasonal effect ranging from ca. 24.4% to 37% was found. This dependency effect makes the complete modelling of a community even more complicated. The required data are difficult to obtain, and unlike in this example, this will frequently happen through cluster borders. However, it is unlikely that the dependency effect is strong enough to reduce the seasonality due to ventilation behaviour to a negligible level.

## Conclusions

Although the real-world “size” of the additional ventilation is difficult to determine, the results suggest that the seasonal change in the ventilation behavior contributes significantly to the overall seasonal effect. Since the differences between very infectious and less infectious diseases are not very extensive, the (relative) effect is probably of a similar magnitude for different airborne diseases. Partial immunities which lower the attack rates will thus probably also not affect it significantly. The additional calculations for dependency suggest that mitigation measures in situations with low expected exposure rates are not very effective for the overall situation.

## Notes

### Competing interests

The author works as a research engineer in the field of aerosol separation at Delbag GmbH (Herne). The manuscript was written independently of this activity, without any company involvement.

### Funding

The author received no financial support for the research, authorship, and/or publication of this article.

### ORCID ID

The author’s ORCID ID is 0000-0003-0745-1106.

## Figures and Tables

**Table 1:**
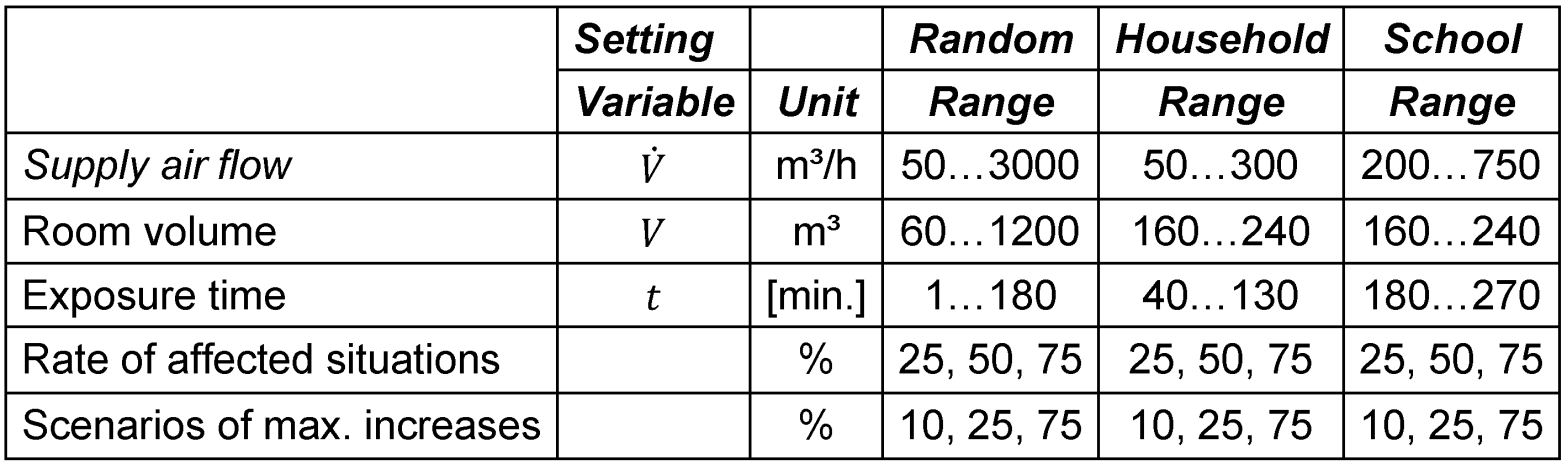
Ranges for randomisation and parametrisation

**Figure 2:**
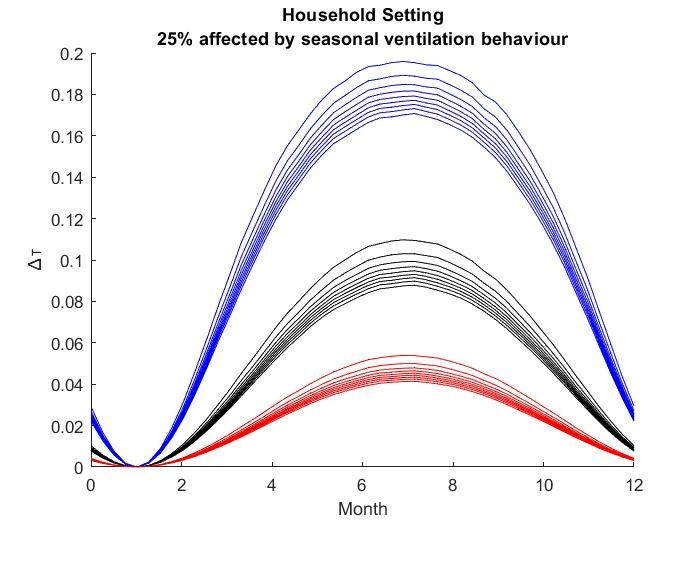
Household Setting with 25% of all situations affected

**Figure 3:**
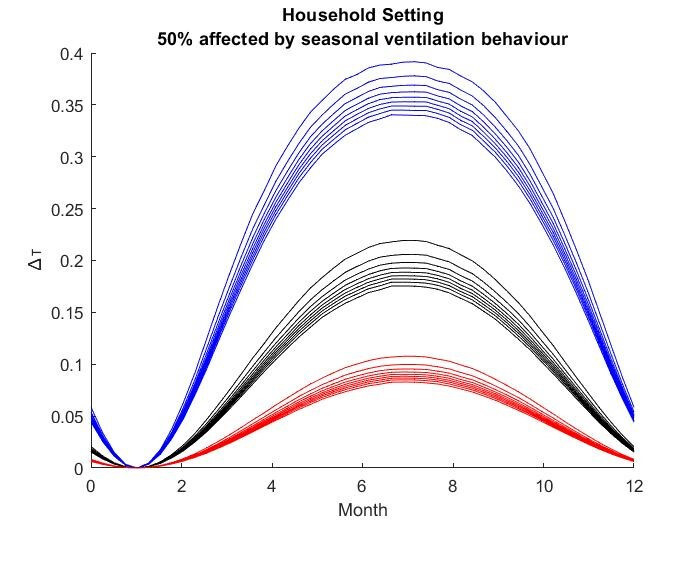
Household Setting with 50% of all situations affected

**Figure 4:**
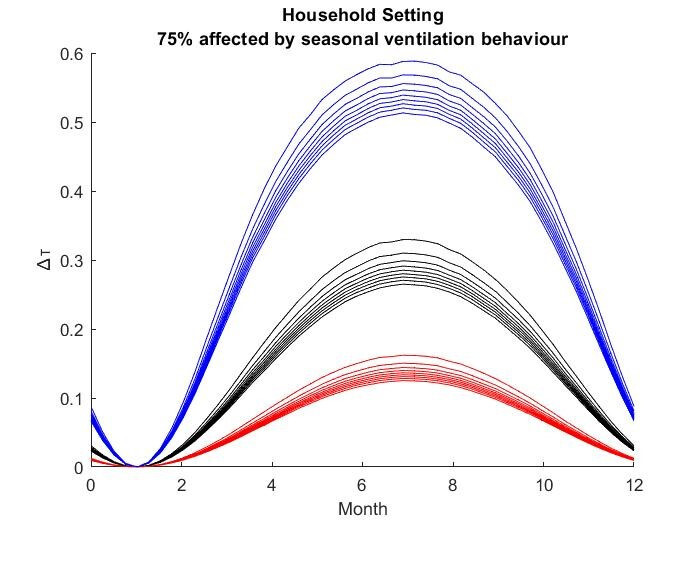
Household Setting with 75% of all situations affected

**Figure 5:**
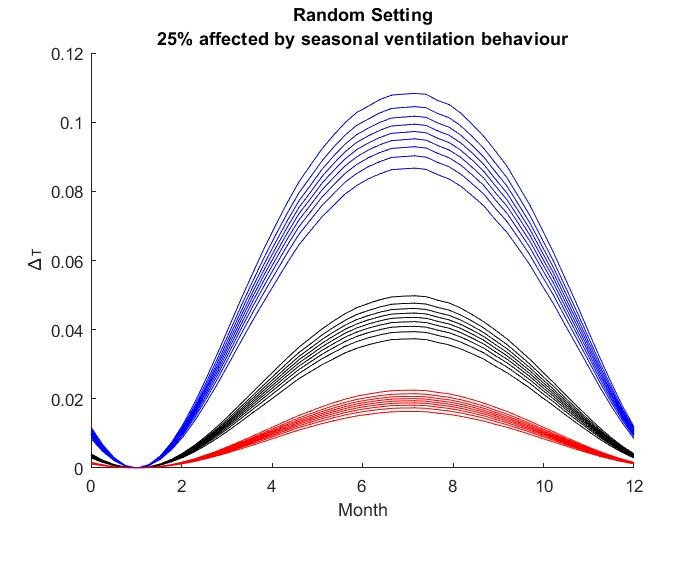
Figure 5 : Random Setting with 25% of all situations affected

**Figure 6:**
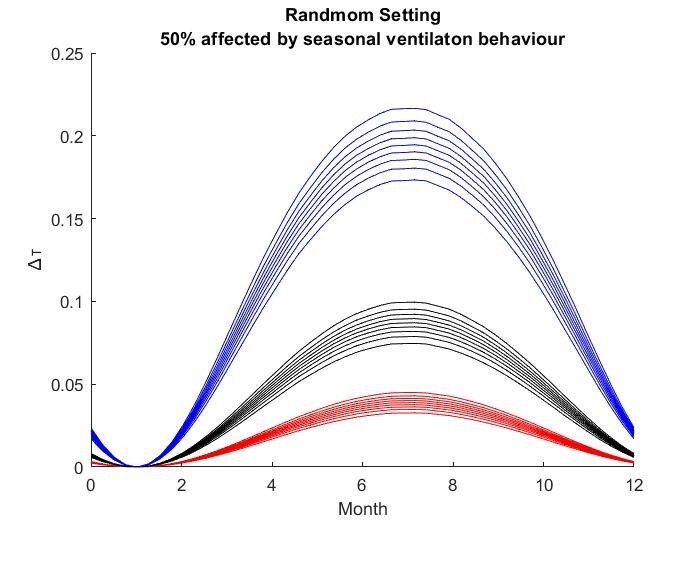
Random Setting with 50% of all situations affected

**Figure 7:**
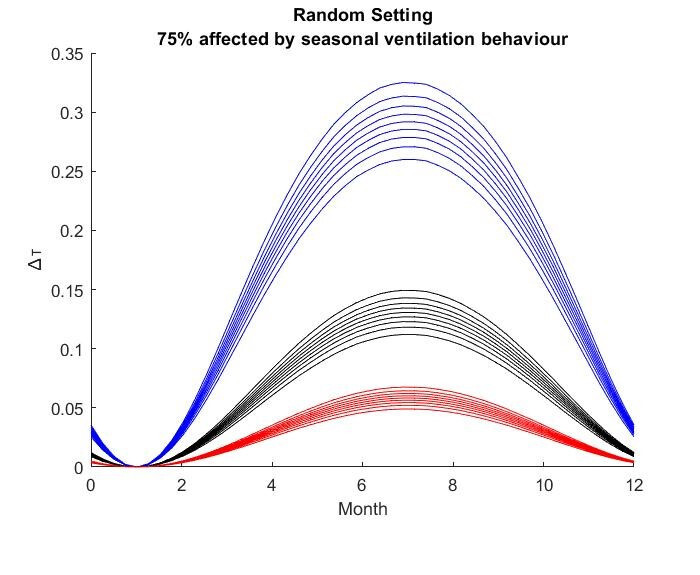
Random Setting with 75% of all situations affected

**Figure 8:**
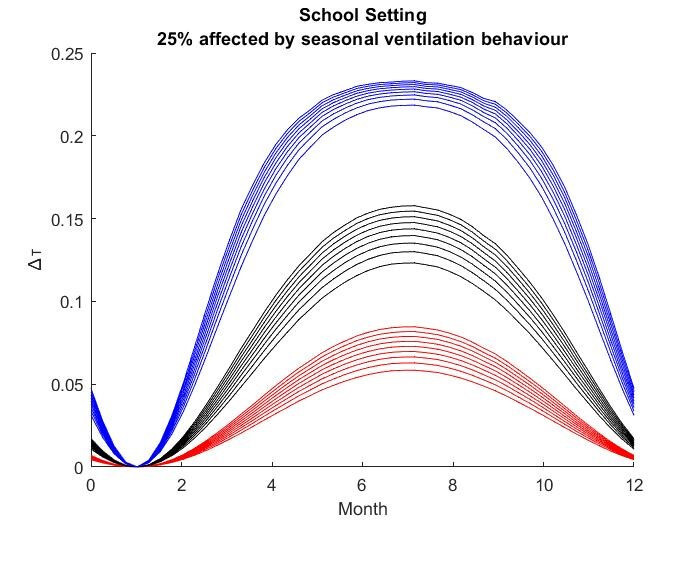
School Setting with 25% of all situations affected

**Figure 9:**
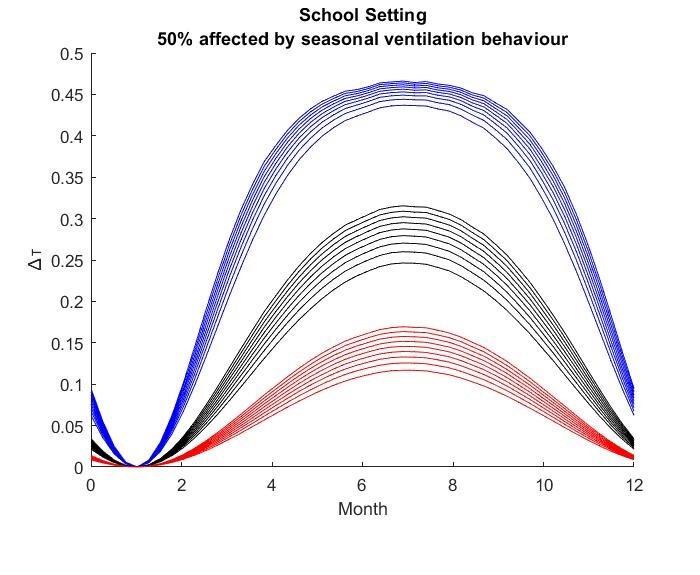
School Setting with 50% of all situations affected

**Figure 10:**
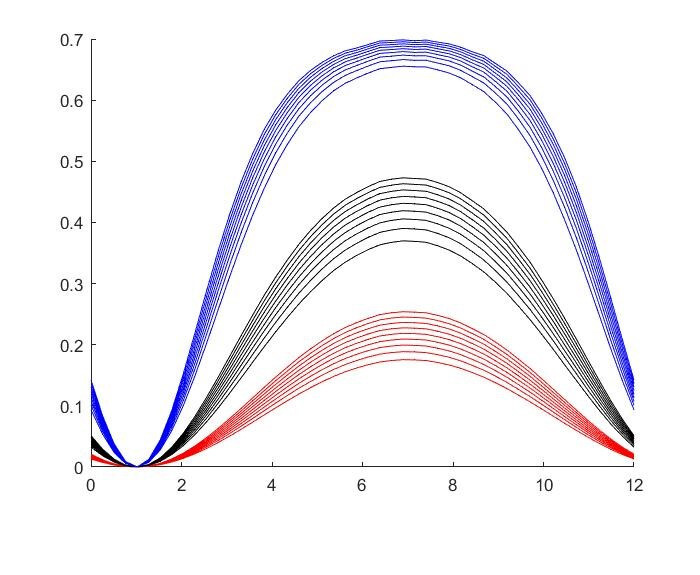
School Setting with 75% of all situations affected
